# Enhanced Cancer Therapy Using an Engineered Designer Cytokine Alone and in Combination With an Immune Checkpoint Inhibitor

**DOI:** 10.3389/fonc.2022.812560

**Published:** 2022-03-24

**Authors:** Anjan K. Pradhan, Praveen Bhoopathi, Santanu Maji, Amit Kumar, Chunqing Guo, Padmanabhan Mannangatti, Jiong Li, Xiang-Yang Wang, Devanand Sarkar, Luni Emdad, Swadesh K. Das, Paul B. Fisher

**Affiliations:** ^1^ Department of Human and Molecular Genetics, Virginia Commonwealth University, School of Medicine, Richmond, VA, United States; ^2^ Virginia Commonwealth University (VCU) Institute of Molecular Medicine, Virginia Commonwealth University, School of Medicine, Richmond, VA, United States; ^3^ Virginia Commonwealth University (VCU) Massey Cancer Center, Virginia Commonwealth University, School of Medicine, Richmond, VA, United States; ^4^ Department of Medicinal Chemistry, Philips Institute for Oral Health Research, Virginia Commonwealth University, School of Pharmacy, Richmond, VA, United States

**Keywords:** secretory motif, *mda-7 (IL-24)*, M7S (IL-24S), insulin signal peptide; PD-L1, melanoma

## Abstract

*melanoma differentiation associated gene-7 or Interleukin-24* (*mda-7, IL-24*) displays expansive anti-tumor activity without harming corresponding normal cells/tissues. This anticancer activity has been documented *in vitro* and *in vivo* in multiple preclinical animal models, as well as in patients with advanced cancers in a phase I clinical trial. To enhance the therapeutic efficacy of MDA-7 (IL-24), we engineered a designer cytokine (a “Superkine”; IL-24S; referred to as M7S) with enhanced secretion and increased stability to engender improved “bystander” antitumor effects. M7S was engineered in a two-step process by first replacing the endogenous secretory motif with an alternate secretory motif to boost secretion. Among four different signaling peptides, the insulin secretory motif significantly enhanced the secretion of MDA-7 (IL-24) protein and was chosen for M7S. The second modification engineered in M7S was designed to enhance the stability of MDA-7 (IL-24), which was accomplished by replacing lysine at position K122 with arginine. This engineered “M7S Superkine” with increased secretion and stability retained cancer specificity. Compared to parental MDA-7 (IL-24), M7S (IL-24S) was superior in promoting anti-tumor and bystander effects leading to improved outcomes in multiple cancer xenograft models. Additionally, combinatorial therapy using MDA-7 (IL-24) or M7S (IL-24S) with an immune checkpoint inhibitor, anti-PD-L1, dramatically reduced tumor progression in murine B16 melanoma cells. These results portend that M7S (IL-24S) promotes the re-emergence of an immunosuppressive tumor microenvironment, providing a solid rationale for prospective translational applications of this therapeutic designer cytokine.

## Introduction

MDA-7, also known as IL-24, is a unique member of the IL-10 gene family, first cloned in our laboratory (1–7) through subtraction hybridization. Today, three decades after cloning, an extensive number of studies in multiple laboratories have demonstrated profound multimodal anti-tumor activities of this therapeutic cytokine including selective induction of cell death/toxic autophagy in cancer cells (1, 4, 8–10), systemic antitumor activity (11, 12), immune modulatory activity (12–15), and anti-angiogenic activity (2, 4, 8, 9, 12, 15–17). Safety and therapeutic efficacy has been confirmed in a Phase I clinical trial in humans with advanced cancers (carcinomas and melanomas) by direct injection into tumors of a replication incompetent adenovirus expressing *mda-7* (INGN 241; Ad.*mda-7*) (18, 19). One notable characteristic of MDA-7-based therapy is its ability to exert a cancer-specific “bystander” antitumor effect, which directly contributes to its profound anticancer properties *in vivo*.

Following Ad.*mda-7* infection MDA-7 is synthesized and secreted, subsequently interacting with cognate receptor pairs, IL-20R/IL-22R, expressed on the surface of neighboring and distant normal and cancer cells. Following this interaction, MDA-7 bound to its receptors are internalized stimulating production of this cytokine through a paracrine/autocrine loop (20–22). This induction leads to MDA-7 synthesis in untreated cells where normal cells show no negative impact, whereas neoplastic cells undergo endoplasmic reticulum (ER) stress and die through apoptosis or toxic autophagy (1–7). Accordingly, efficient secretion is a prerequisite for enhanced MDA-7-mediated anticancer outcomes, particularly in the context of metastatic lesions.

Protein secretion encompasses the processes of gene transcription, mRNA translation, protein folding and membrane trafficking (23). As observed with growth factors and other secretory proteins, the secretion of cytokines is tightly controlled by intracellular secretory pathways (23). Most cytokines possess a signal peptide (SP), also referred to as a leader sequence, at their N-termini that guide them to the secretion pathway across the ER membrane to the Golgi compartment and to the cell surface for secretion (23). Ultimately, cytokines are released from cells either constitutively or upon specific stimulation. The prevailing hypothesis is that secretion variability of proteins is determined by the signal peptide (23). Based on this consideration, it is theoretically possible to alter the secretory patterns of proteins by engineering these proteins with unnatural or engineered signal peptides to enhance protein secretion. Key factors also involved in protein secretion involve the secondary structure of the protein and rates of transcription of the secretory protein (23). Considering these factors, we presently sought to develop a strategy to enhance the anticancer “bystander” activity of MDA-7. This was accomplished by enhancing both the secretion and stability of MDA-7 using genetic engineering approaches that included replacing the endogenous SP with an unnatural SP (enhancing secretion) and mutation of the MDA-7 protein at amino acid 122 (24) to inhibit proteasomal degradation (increasing stability). To evaluate the preclinical properties of this engineered designer cytokine (an “MDA-7 Superkine”; M7S) it was packaged into a type 5 adenovirus (Ad.5-*M7S*).

Our expanded understanding of immune tolerance of tumor cells has culminated in the development of multiple effective therapeutic approaches against solid cancers from different histological origins (25). Tumor cells achieve immune tolerance through multiple well-characterized mechanisms including induction of autochthonous tumors, creating an immunosuppressive microenvironment, and activating negative regulatory pathways causing T cell exhaustion (26). Programmed death ligand 1 (PD-L1) is expressed on tumor cells in response to inflammatory factors secreted by tumor-microenvironmental resident cells. PD-L1 binds with PD-1 on T cells resulting in suppression of T cell activation, promoting selective survival of tumor cells. The consequence of blocking the immune checkpoint molecule PD-1 or its ligand PD-L1 using antibodies has become a game-changer in the treatment of some advanced stage diseases (27). Preclinical studies confirm the relevance of PD-1 and PD-L1 in regulating immune responses (28, 29). Positive therapeutic outcomes have been observed with increased success rates following anti-PD-L1 therapy, particularly in melanoma (28). While single agent, i.e., α-PD-L1 remains a viable therapy, a significant proportion of patients remain poorly responsive to this therapy (30). These subsets of patients may benefit from a combinatorial approach that includes anti-PD-L1 therapy with a second therapy.

An additional attribute of MDA-7/IL-24, and perhaps the one most applicable from a translational perspective is the ability to synergize with standard of care therapeutics, e.g., radiation and chemotherapy, promoting cancer cell death (6, 9). Potential augmented therapeutic activity with immune checkpoint inhibitors also represent a practical strategy to successfully enhance treatment of advanced cancers. We demonstrated previously that intratumorally delivery of MDA-7 (by means of a replication-incompetent Ad.5*-mda-7)* stimulated systemic antigen-specific CD8^+^ T cell responses in breast cancer (14), supporting an immunological contribution of MDA-7 in mediating breast cancer killing. A recent study describes potential synergy between anti-PD-1 and an armed replicating adenovirus expressing wild type *mda-7* in B16 mouse melanoma cells *in vivo* in syngeneic mice (31). In the present study, we demonstrate enhanced therapeutic potential *in vitro* and *in vivo* of M7S vs. parental MDA-7 against multiple human tumors (melanoma, prostate and breast) and murine B16 melanoma tumors when delivered by a replication incompetent adenovirus (Ad.5-*M7S*) and the antitumor effect is further enhanced when combined with α-PD-L1 in the aggressive B16 melanoma model.

## Materials and Methods

### Cell Lines

Cell lines from different anatomic origin were used in this study. These included human melanoma (MeWo, RPMI-7951), human prostate (DU-145, PC3-ML) and human breast (MDA-MB-231, MCF-7) cancer cell lines that were obtained from the ATCC, and maintained in their recommended culture media. MCF10A, a non-tumorigenic epithelial cell line was also obtained from ATCC. Cell lines were routinely tested for mycoplasma by a PCR approach (Sigma-Aldrich, USA). Immortalized normal human melanocytes FM-516-SV (32) and normal human prostate epithelial cells RWPE-1 (8) were used as primary control normal cells. FM-516-SV cells were obtained from Dr. Leila Diamond (Wistar Institute, PA) and cultured as previously described (33). RWPE-1, is a human papillomavirus 18 immortalized adult normal human prostate cell line obtained from the ATCC and maintained in keratinocyte serum-free medium (K-SFM) (Gibco) supplemented with 0.05 mg/ml BPE and 5 ng/ml EGF (34). Murine melanoma, B16 cells, were obtained from the ATCC and cultured as described previously (35).

### Cloning and Adenovirus Generation

Different secretion motifs as described in this study were cloned in the N’ region of *mda-7* (*IL-24*) in a pcDNA3.1 vector. Clones were validated by DNA sequencing. The Ad clones expressing *mda-7* and *mda-7* with an insulin secretory motif and a mutation (lysine 122 to arginine) of the MDA-7 protein at amino acid 122, Ad.5-*M7S*, were constructed using standard cloning procedures (3). Ad amplification, purification, titration, and infection were performed using standard protocols (3). Adenoviruses were purified using the cesium chloride density gradient method. Titers were assessed by PFU (plaque-forming unit) and by measuring infectious viral particles (VP). Cells were infected with the Ads (expressing the gene of interest or a control Ad.5-*null*) in serum-free cell culture medium. After 2 hr., complete media was added, and cells were incubated at 37°C in a humidified incubator for different times.

### *In Vivo* Experiments

All animal studies were conducted following the University IACUC Policies under an approved protocol. Human tumor xenografts (MeWo and DU-145) were established in both the right and left flank of 8-week-old male athymic nude mice, purchased from Envigo laboratories (Envigo, USA). Two-month-old C57BL/6 mice were also purchased from the same vendor and used for B16 murine melanoma study.

### RNA Isolation and RQ-PCR

Real time quantitative PCR was performed on cDNA prepared (Applied Biosystems, USA) from total RNA isolated with the RNA isolation kit Qiagen (Valencia, CA, USA). TaqMan probes and the Real time 2X master mix were from Applied Biosystems (Foster City, CA, USA). Data were analyzed and graphically represented with Graph pad prism software.

### Western Blotting

SDS-PAGE and Western blotting was performed using previously established protocols (36). The primary antibodies used were MDA-7 (IL-24) (Genhunter, Nashville, TN, USA), and Actin and PARP (Cell Signaling Technology, Boston, MA, USA). Secondary antibodies were purchased from Cell Signaling Technology (Boston, MA, USA). MDA-7 (IL-24) ELISA kit was from R&D systems, MN, USA. Antibodies used were CD3, CD4, CD8, IFN-γ, CD45, and PD-L1 (Biolegend, San Diego, CA, USA). Therapeutic PD-L1 antibody and the Control antibodies were from Bio X Cell (West Lebanon, NH, USA).

### Flow Cytometry-Based Protein Expression

For membrane protein expression and intracellular staining, cells were washed and stained with Alexa-Fluor tagged antibodies. Fluorescence signals were measured with BD FACS Canto flow cytometer and BD Fortessa flow cytometer (BD Biosciences, San Jose, CA). Data were analyzed with FACs DIVA software (BD Biosciences). Antibodies used included CD3, CD4, CD8, IFN-γ, CD45, and PD-L1 (Biolegend, San Diego, CA, USA). Therapeutic PD-L1 antibody and control antibodies were from Bio X Cell (West Lebanon, NH, USA).

### Immunohistochemistry

At the termination of the animal experiments, mice were sacrificed, and the tumor tissues were preserved in 10% formalin. Tissue sections (5 µm) were prepared and processed for immunohistochemistry as described previously (8). The following antibodies were used for IHC, Ki-67 antibody (Vector laboratories, USA) and MDA-7 (IL-24) antibody (Genhunter, Nashville, TN, USA). Images were analyzed microscopically at a magnification of 40X.

### Tunnel Assay

Formalin-fixed and paraffin-embedded 5 µm tissue sections were processed with DeadEnd™ Fluorometric TUNEL System (Promega, Madison, WI, USA) according to the manufacturer’s protocol. Apoptotic cells (green) and DAPI-positive (blue) cells were imaged and analyzed with an inverted fluorescence microscope at a magnification of 40X.

### Cell Proliferation Assays

Cell proliferation was quantified using a standard MTT assay as described previously (21).

### Statistical Analysis

Unless specified, all *in vitro* and *in vivo* data were analyzed using Graph Pad Prism. Student’s T tests were performed to determine the statistical significance between two variables. Probability value less than 0.05 is considered significant.

## Results

### Augmenting the Unique Anticancer Properties of MDA-7 (IL-24) Through Genetic Engineering: Creating a Designer *mda-7 (IL-24)* Cytokine (“MDA-7 Superkine”; IL-24S; M7S) With Enhanced Secretion and Stability

**Figure 1:**
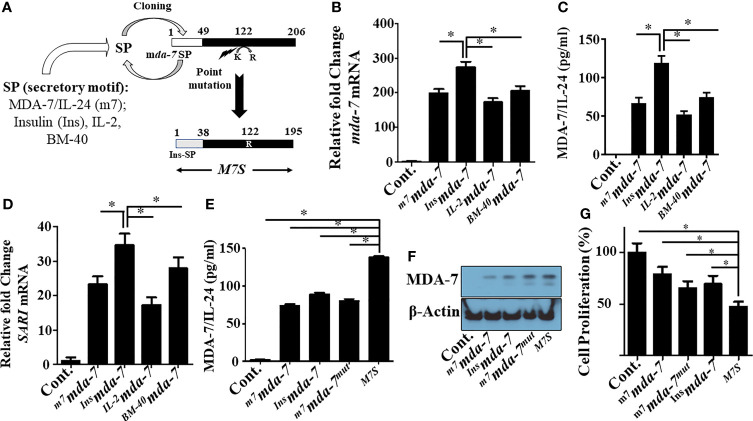
Engineering and primary characterization of a designer cytokine, “MDA-7/IL-24 Superkine” (M7S; IL-24S). **(A)** Schematic illustration of the creation of an “MDA-7/IL-24 Superkine” (M7S; IL-24S) protein (Not drawn to scale). The endogenous signal peptide sequence of MDA-7/IL-24 was replaced with a secretory peptide motif from the corresponding proteins (as indicated) to define secretion capability (tested in panel **B–D**. An additional point mutation (Lysine to Arginine) was introduced and combined with the Insulin signal peptide (Ins^SP^) carrying *mda-7* to enhance protein stability. The resulting mutant construct with both changes is called a “designer cytokine”. **(B)** DU-145 cells were transfected with the indicated mutants and the conditioned media (CM) from transfected cells were collected and normalized, based on total protein expression. DU-145 cells were incubated with CM supplemented culture media for 24 hr. and the transcript levels of *mda-7/IL-24* were determined with q-PCR. Fold changes of *mda-7/IL-24* are maximum in cells treated with CM from Insulin-SP-*mda-7* (*^Ins^mda-7*) transfected cells. **(C)** DU-145 cells were incubated with CM isolated from different experimental plasmid-transfected cells as described in Panel **(B)** Quantification of expression of secretory MDA-7/IL-24 protein in the culture media was determined by ELISA. **(D)** Fold-change in the mRNA transcript level of *SARI*, a downstream marker of *mda-7/IL-24* treatment, in DU-145 cells treated with CM isolated from DU-145 cells transfected with the indicated plasmids. Fold-change of up regulation of SARI (mRNA) is maximum in cells treated with CM from *^Ins^mda-7* transfected cells. **(E, F)** Expression of MDA-7/IL-24 protein in the CM of DU-145 cells following transfection with the indicated plasmids as determined by ELISA and Western blotting. **(G)** MTT assays showing inhibition of cellular proliferation in DU-145 cells treated with the CM isolated from DU-145 cells transfected with the indicated plasmids. *Statistical significance (p < 0.05).

MDA-7 embodies multiple properties supporting its use as an effective cancer gene/protein therapeutic (7). The bioactivity of purified MDA-7 protein toward tumor cells is cell surface receptor- and concentration-dependent, and direct cancer cell growth suppression and killing occurs only when a complete set of dimeric receptors are present, and a threshold level of this cytokine is achieved (22). Accordingly, since MDA-7 is a secreted protein that mediates many of its tumor suppressive functions through cell surface receptors, we endeavored ways to enhance secretion. To achieve this objective, we evaluated three unnatural signal peptides linked to MDA-7 *vs*. the endogenous signal peptide present in MDA-7. The four signal peptides (SP) tested were: endogenous human MDA-7, human IL-2, human Insulin, and human BM40 (osteonectin/SPARC) SPs (outlined in **Figure 1A**). Comparative activity was determined based on the documented ability of this cytokine to induce autocrine/paracrine signaling, in which exogenous secreted MDA-7 protein can induce endogenous MDA-7 mRNA and protein in receptor positive cells through an autocrine mechanism (8, 21, 37). Accordingly, *mda-7* transcript levels were measured as a functional readout of MDA-7 receptor engagement. As shown in **Figure 1B**, *mda-7* containing the insulin SP (*^Ins^mda-7*) maximally increased the level of *mda-7* transcripts.

The engineered cytokine constructs were transfected into DU-145 (prostate cancer) cells and ELISA measured the amount of MDA-7 protein in the conditioned media (**Figure 1C**). The construct with the insulin SP was again the most effective designer cytokine in enhancing MDA-7 protein secretion vs. the other 3 SPs. We also confirmed its functionality *in vitro* through induction of *SARI* mRNA, an MDA-7/IL-24 downstream responsive gene (21) (**Figure 1D**). *SARI* upregulation was again greatest in *^Ins^mda-7* transfected cells **(** **Figure 1D** **)**. In total, these results show that the insulin secretion motif enhances the secretion of MDA-7 protein to a greater extent than the endogenous *mda-7*, IL-2 or BM-40 SPs.

Tian and colleagues suggested that mutating lysine at position 123 to arginine could enhance MDA-7 protein stability (24). Analysis of the protein sequence of the originally cloned MDA-7 gene identified in our laboratory (NM_006850.3) indicated that lysine was at position 122 and not position 123. We mutated lysine 122 to arginine by site-directed mutagenesis (K122R) and combined this with the insulin (sp)-*mda-7* creating the next generation “MDA-7 Superkine” (M7S; ^Ins^M7^mut^). We next determined the presence of MDA-7 secreted protein in the conditioned media (**Figure 1E**) and the intracellular (**Figure 1F**) level in DU-145, transfected with full-length *mda-7* vs. various engineered versions of this cytokine. This experiment documented an increase in MDA-7 protein when containing an insulin secretory motif and a point mutation. To determine if enhanced secretion is a general property of the insulin signal peptide when linked to other secreted proteins, we engineered IL-2 to contain this peptide, *^Ins^IL-2*, and evaluated secretion in comparison with IL-2 containing its endogenous SP (**Supplementary Figures 1A, B** **)**. Unexpectedly, in the context of IL-2, secretion was reduced when the endogenous IL-2 secretory peptide was replaced with the insulin secretory peptide. These results highlight the complexity of protein secretion that involves both the secretory motif as well as the inherent structure of the secreted protein. As shown previously (21), MDA-7/IL-24 inhibits the proliferation of cancer cells and of the engineered molecules tested, M7S maximally inhibited growth as measured by MTT assays **(** **Figure 1G** **)**.

### Developing Adenoviruses Expressing Designer Cytokines (“MDA-7 Superkine (IL-24S; M7S); Ad.5-*M7S*)

**Figure 2:**
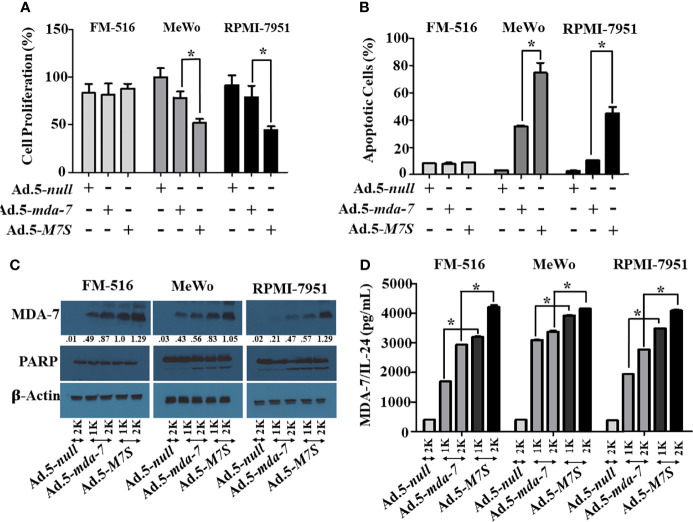
Ad.5-*M7S* suppresses proliferation and induces apoptosis of melanoma cells more robustly than wild type Ad.5-*mda-7.* **(A, B)** Immortal primary melanocytes (FM-516) and melanoma cell lines (MeWo and RPMI-7951) were infected with Ads expressing either *mda-7/IL-24* or *M7S* at different m.o.i. After 72 hr., cell proliferation **(A)** and apoptosis **(B)** were determined by MTT assay and Flowcytometry (Annexin-V/PI- staining), respectively. **(C)** Western blotting analysis was performed to determine intracellular MDA-7/IL-24 protein levels and PARP cleavage (an indicator of apoptosis). Actin was used as a loading control. Values underneath the blot represent fold-changes in densitometry for MDA-7/IL-24 protein levels using actin as a control. **(D)** Secretion of MDA-7/IL-24 was determined in infected cell-derived CM using MDA-7/IL-24-specific ELISA. *Statistical significance (p<0.05). Different virus dose indicated as viral particles (VP): 1K: 1000 VP; 2K: 2000 VP.

**Figure 3:**
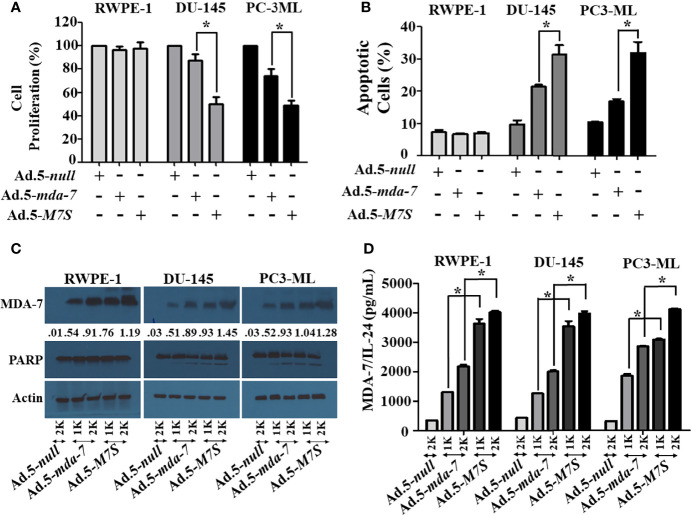
Ad.5-*M7S* suppresses proliferation and induces apoptosis of prostate cancer cells more robustly than wild type Ad.5-*mda-7.* **(A, B)**. Different prostate cancer cells (DU-145 and PC3-ML), and immortal primary prostate epithelial cells (RWPE-1) were infected with Ads expressing either *mda-7/IL-24* or *M7S* (*IL-24S*) at different m.o.i. After 72 hr., cell proliferation **(A)** and apoptosis **(B)** were determined by MTT assay and Flow-cytometry (Annexin-V/PI- staining), respectively. **(C)** Western blotting analysis was performed to determine intracellular MDA-7/IL-24 protein levels and PARP cleavage. Actin was used as a loading control. Values underneath the blot represent fold-changes in densitometry for MDA-7/IL-24 protein levels using actin as a control. **(D)** Secretion of MDA-7/IL-24 was determined in infected cell-derived CM using MDA-7/IL-24-specific ELISA. *Statistical significance (p<0.05). Different virus dose indicated as viral particles (VP): 1K: 1000 VP; 2K: 2000 VP.

**Figure 4:**
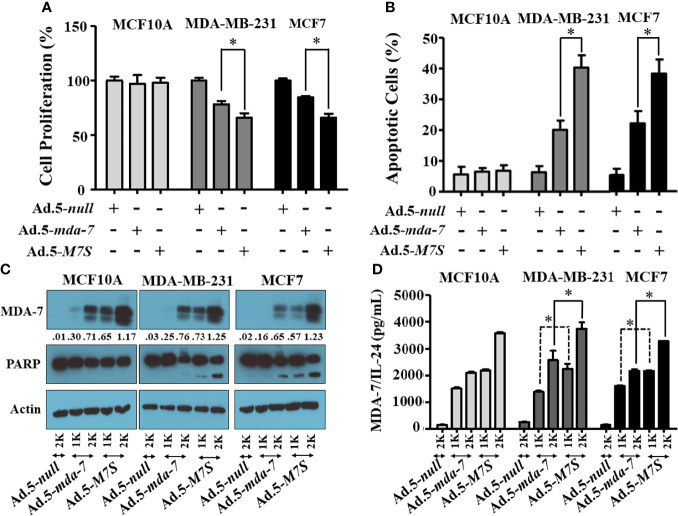
Ad.5-*M7S* suppresses proliferation and induces apoptosis of breast cancer cells more robustly than wild type Ad.5-*mda-7/IL-24*. A & B) Indicated cells were infected with Ads expressing either *mda-7/IL-24* or *M7S* (*IL-24S*) at different m.o.i. After 72 hr., cell proliferation **(A)** and apoptosis **(B)** were determined by MTT assay and Flow-cytometry (Annexin-V/PI- staining), respectively. **(C)** Western blotting analysis was performed to determine intracellular MDA-7/IL-24 protein levels and PARP cleavage. Actin was used as a loading control. Values underneath the blot represent fold-changes in densitometry for MDA-7/IL-24 protein levels using actin as a control. **(D)** Secretion of MDA-7/IL-24 was determined in infected cell-derived CM using MDA-7/IL-24-specific ELISA *: Statistical significance (p < 0.05). Different virus dose indicated as viral particles (VP): 1K: 1000 VP; 2K: 2000 VP.

Our fundamental hypothesis is that M7S will display greater antitumor activity against cancer cells than native *mda-7*. To facilitate this comparison and to provide greater accuracy than is possible by plasmid transfection, we developed a non-replicating Ad.5 (Adenovirus serotype 5) in which *M7S* is under the transcriptional control of a CMV promoter (Ad.5-*M7S)*, like Ad.5-*mda-7*. Multiple cell lines including both normal and cancer cells from different cancer types including human melanoma (**Figure 2**), prostate (**Figure 3**) and breast (**Figure 4**) were infected with Ads carrying wild type *mda-7* or *M7S* and effects on cell proliferation (MTT assays) were evaluated (**Figures 2A**, **3A** and **4A**). Expression of both versions of MDA-7 inhibited growth of the diverse cancer cell lines (p<0.05 vs. control) without showing any detrimental effects on corresponding immortal normal human cell lines (**Figures 2A**, **3A** and **4A**). Ad.5-*M7S* demonstrated enhanced anti-proliferative effects in all cancer cell lines vs. Ad.5-*mda-7*. Moreover, as reported with other human cancer cells (8, 21, 37), Ad.5-*M7S*-mediated proliferation suppression was a consequence of induction of apoptosis (**Figures 2B**, **3B** and **4B**). Infection of tumor cells with Ad.5-*M7S* resulted in a significantly higher level of secreted protein in culture media in comparison with wild type Ad.5-*mda-7*. This was observed both in cell lysates and conditioned media as documented by Western blotting **(** **Figures 2C**, **3C** and **4C** **)** and ELISA **(** **Figures 2D**, **3D** and **4D** **),** respectively. Also, cellular lysates had increased cleavage of PARP in Ad.5-*M7S*-infected cells as compared to Ad.5-*mda-7-*infected cells **(** **Figures 2C**, **3C** and **4C** **)**. These results document that in addition to enhanced *in vitro* antitumor activity, Ad.5-*M7S* retains selective anticancer activity in human cancer cells as observed previously in different cancer/normal contexts with Ad.5-*mda-7* (5).

**Figure 5:**
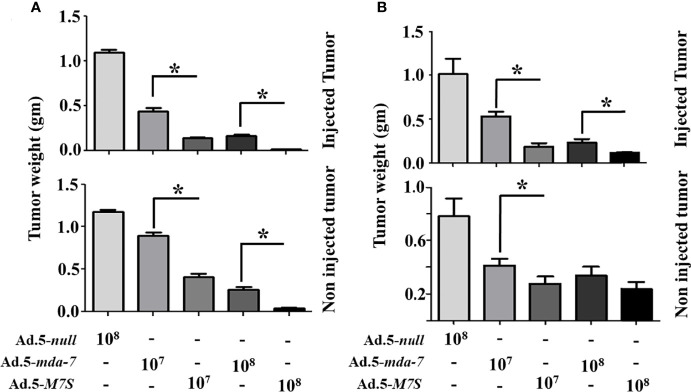
M7S (IL-24S) displays potent *in vivo* bystander activity. **(A)** 5X10^6^ MeWo cells (melanoma cancer cell line) were injected subcutaneously into both flanks of nude mice. Once palpable tumors formed (~100 cc), Ad.5-*null*, Ad.5-*mda-7* or Ad.5-*M7S* was administered intratumorally (3X in a week, 6 injections in total). Mice were sacrificed on day 21 after the first viral injections. Tumor weight was measured. Data presented as the average weight at day 14 from 5 mice from each group. **(B)** Like panel **(A)**, DU-145 cells (prostate cancer cell line) were implanted in nude mice and infected with the indicated control virus (Ad.5-*null*) and therapeutic viruses (Ad.5-*mda-7* or Ad.5-*M7S*). Mice were sacrificed on day 21 after the first viral injections. Average tumor weight after 6 injections is presented. In both **(A, B)**, the “upper” and “lower” panels represent tumors with virus or without virus treatment, respectively. Error bars are S.D. from five mice. *Statistical significance (p < 0.05).

**Figure 6:**
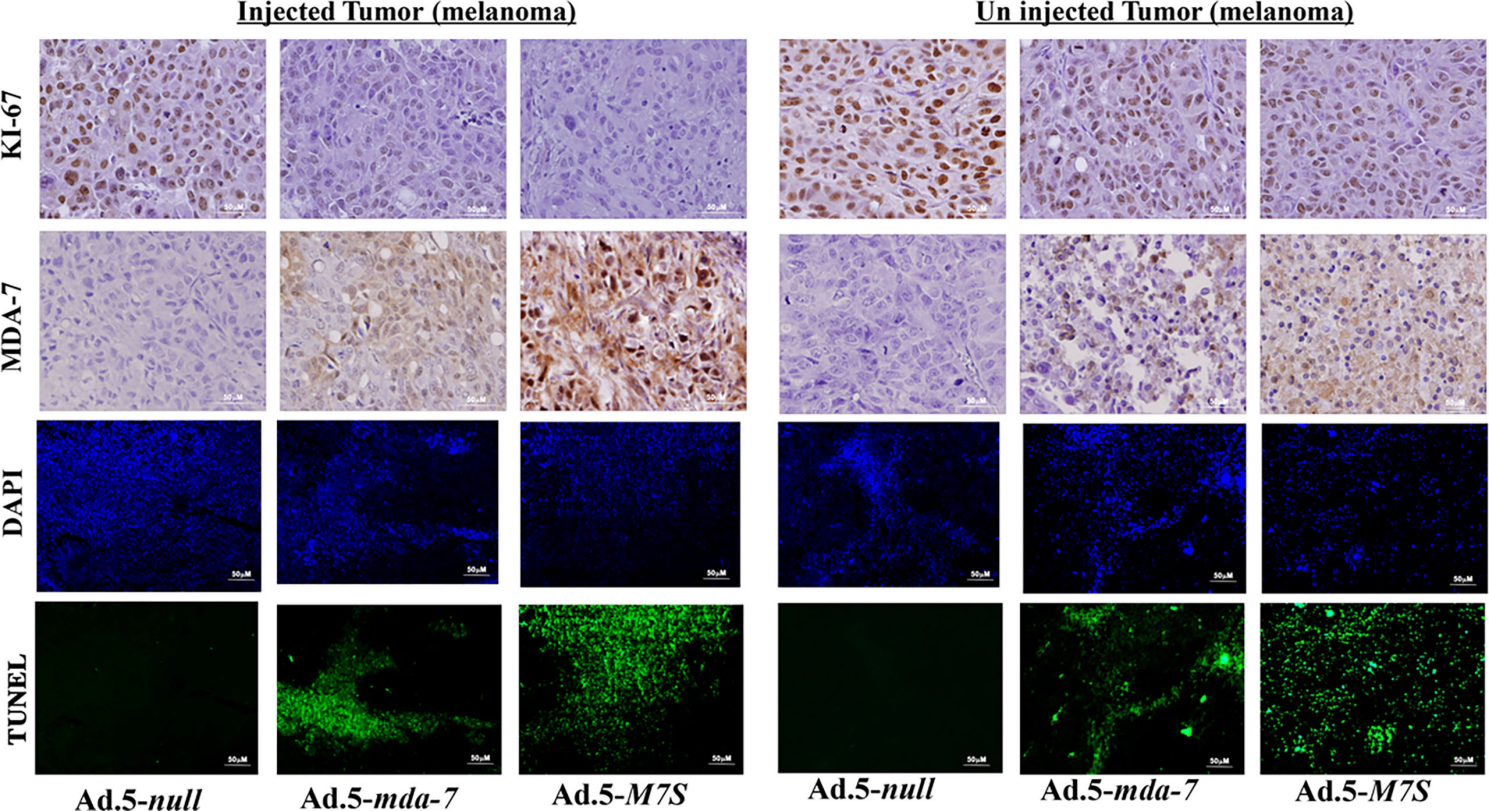
Immunohistochemical analysis of melanoma xenograft tumors injected with Ad.5-*mda-7* or Ad.5-*M7S*. At the end of the experiment, tumor tissues were excised and fixed in 10% formalin. IHC analysis of MDA-7/IL-24 and Ki-67 in representative tumor sections from the melanoma xenograft tumors (from **Figure 5A**) are shown. Sections were immuno-stained by TUNEL to determine apoptosis and DAPI for nuclear staining. Representative photographs are presented from each experimental group. Left panels show the injected tumors, while the right panels show the un-injected tumors.

**Figure 7:**
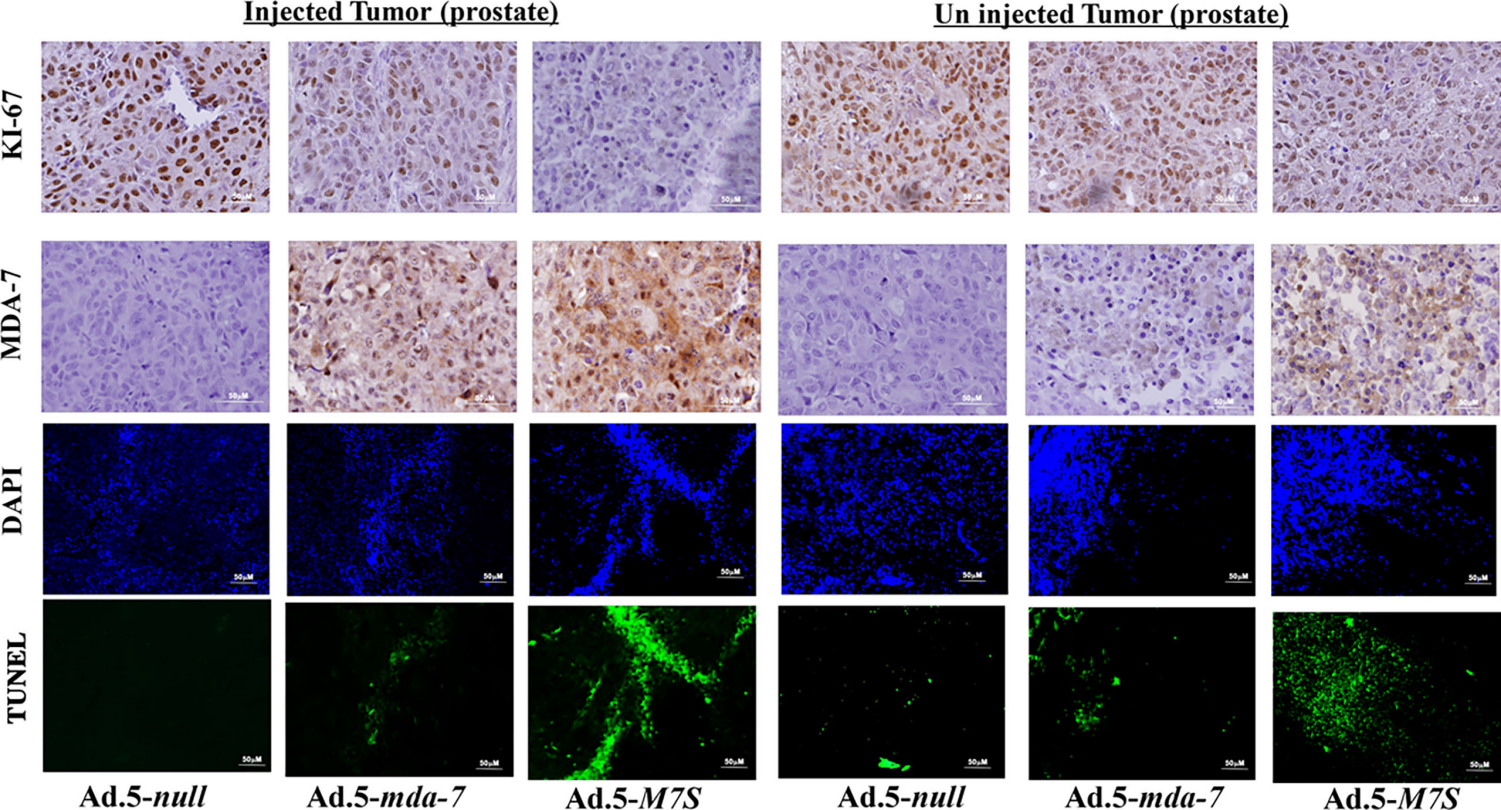
Immunohistochemical analysis of prostate cancer xenograft tumors injected with Ad.5-*mda-*7 or Ad.5-*M7S*. IHC analysis of MDA-7/IL-24 and Ki-67 in the different tumor sections from melanoma xenograft tumors (from **Figure 5B**) are as shown. Sections were also immunostained with by TUNEL to determine apoptosis and DAPI for nuclear staining. Representative photographs are presented from each experimental group. Left panels show the injected tumors while the right panels show the un-injected tumors.

To compare *in vivo* activity, melanoma and prostate cancer tumor xenografts were established on both flanks of athymic nude mice with human melanoma (MeWo; **Figure 5A**) and prostate cancer (DU-145; **Figure 5B**) cells. Ad.5-*mda-7* or Ad.5-*M7S* were injected at different m.o.i. into tumors on the left flank and the right flank did not receive any virus. After 21 days, animals were euthanized and tumors were collected. Both Ad.5-*mda-7* and Ad.5-*M7S* strongly suppressed the growth of the injected tumor (Upper panel **Figures 5A, B** and **Supplementary Figures 2A, B**) and also inhibited growth of the right side contralateral un-injected tumor (Lower panel **Figures 5A, B** and Lower panel **Supplementary Figures 2A, B**). As evident from our *in vitro* experiments, Ad.5-*M7S*-mediated antitumor effects were superior to Ad.5-*mda-7* in both cancer types. This effect correlated with a higher apoptotic index as defined by TUNEL assays and a lower proliferation index as indicated by a decrease in expression of Ki- 67 in treated tumors. Once again, the potency of Ad.5-*M7S* exceeded that observed with Ad.5-*mda-*7 (**Figures 6** and **7**, for melanoma and prostate cancer xenografts, respectively).

### MDA-7 and M7S Synergize With Immune Checkpoint Inhibitors in Suppressing B16 Murine Melanoma Growth in Syngeneic Animals

**Figure 8:**
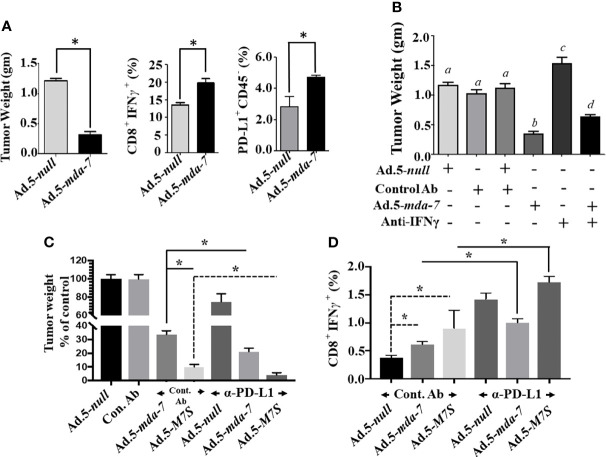
Ad.5-*M7S* and anti-PD-L1 synergistically suppress murine B16 melanoma xenograft growth. **(A)** (Left Panel), Effect of MDA-7/IL-24 on B16 tumor growth in C57BL/6 animals. 5 X 10^5^ B16 cells were injected subcutaneously in C57BL/6 mice and infected with Ad.5-*null* or Ad.5-*mda-7* (intratumoral injection 10^8^ viral particles, 3X in a week, 6 injections in total). Tumor weight was measured. Data presented is the average weight on day 14 from 5 mice from each group. Middle Panel, Cytotoxic T cell accumulation in the tumor. Right panel, PD-L1 expression in the cells isolated from tumors treated with Ad.5-*mda-7*. Immunostaining protocol is described in Materials and Methods. **(B)** Effect of IFN-γ neutralization on the therapeutic effect of *mda-7* on B16 tumor growth. Experimental protocols are described in Materials and Methods. Different letters among variables indicate statistical significance. **(C)** Effect of combinatorial therapy of Ad.5-mda-7 or Ad.5-M7S plus anti-PD-L1 on B16 tumor growth. 5 X 105 B16 cells were injected subcutaneously in C57BL/6 mice and infected with Ad.5-null or Ad.5-mda-7 or Ad.5-M7S (intratumoral injection 108 viral particles, 3X in a week, 6 injections in total). For Anti-PD-L1, 100 μg of antibody was injected in PBS through the intraperitoneal route (3X in a week, 6 injections in total). Mice were sacrificed on day 14 after the first therapeutic injections. Tumor weight was measured. Data presented is the average weight from 5 mice from each group. **(D)** CD8^+^IFN-γ expression in tumors are shown. *Statistical significance (p < 0.05).

**Figure 9:**
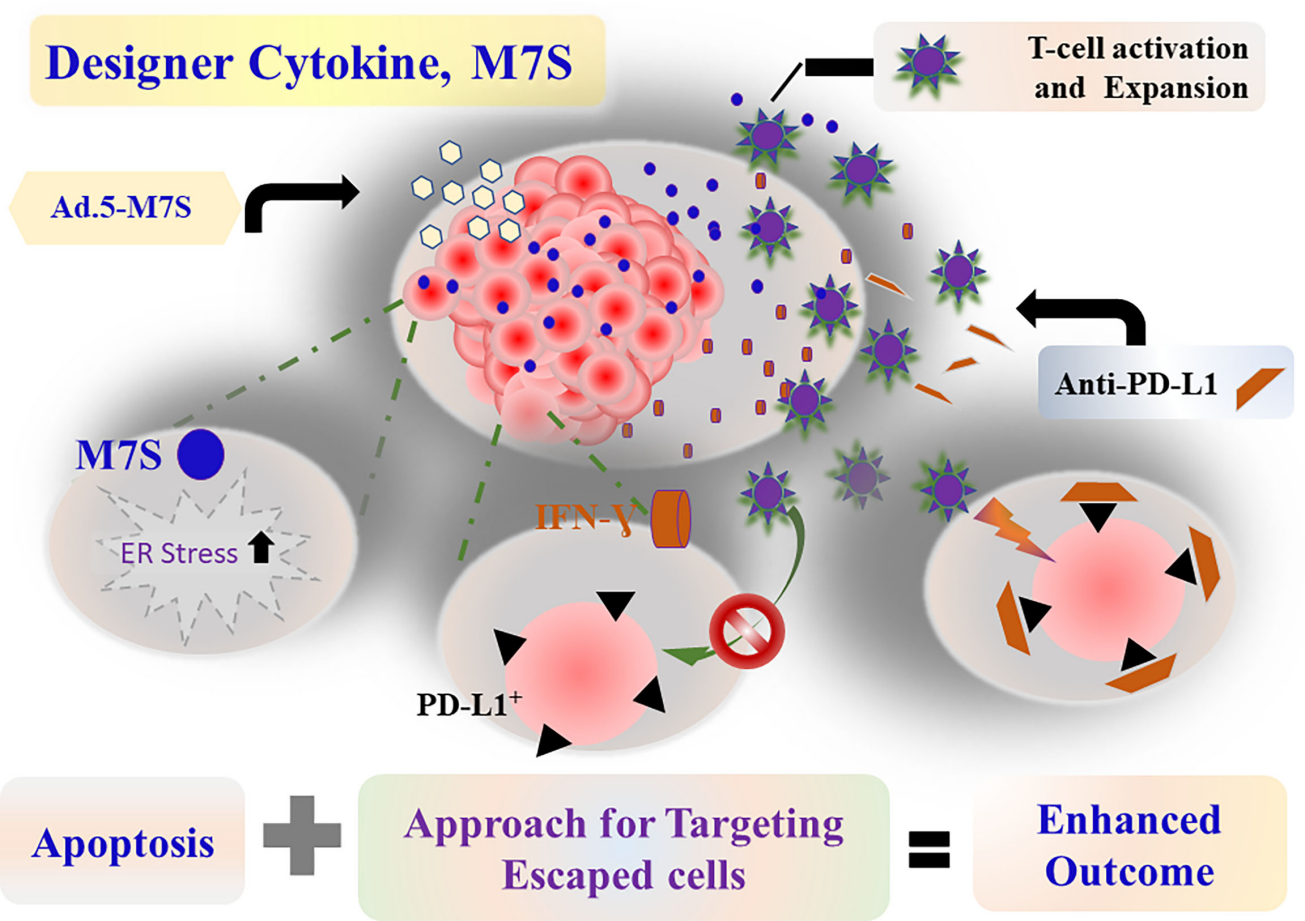
Schematic presentation of MDA-7 (IL-24) action in combination with anti-PD-L1.

The murine B16 melanoma model was used to investigate the effects of Ad.5-*mda-7* and Ad.5-*M7S* on *in vivo* tumor growth in a syngeneic animal model with an intact immune system. After 2 week, animals were sacrificed and tumors were collected. Human MDA-7 did not induce appreciable growth suppression or apoptosis in B16 cells *in vitro* (**Supplementary Figure 3**) in comparison with DU-145 cells. This was not a consequence of lack of viral infectivity or expression of the MDA-7 transgene following infection with Ad.5-*mda-*7 (**Supplementary Figure 3**). In contrast, the tumor regression effect was robust in B16 tumors, signifying that Ad.5-*mda-7* could alter other pathways involved in B16 tumor growth (**Figure 8A**, first panel and **Supplementary Figure 4A**). One possible pathway could involve activation of T cell-mediated killing. This hypothesis was tested and we observed melanoma antigen specific T cell activation, represented by a higher accumulation of IFN-γ-expressing CD8^+^ T cell populations (**Figure 8A**, second panel), in the tumor after infection with Ad.5-*mda-7*. However, we also observed a significant up-regulation of PD-L1^+^CD45^-^ populations in surviving tumor cells, possibly reflecting tumor cells that escaped from immune-mediated killing (**Figure 8A**, third panel). Previous studies demonstrate that IFN-γ induces PD-L1 expression (38). In a different study, it was also suggested that MDA-7 could induce IFN-γ through an unknown mechanism (14, 39). To explain these two opposing phenomena, we hypothesized that MDA-7/IL-24*-*induced IFN-γ upregulated PD-L1 expression in B16 tumor cells resulting in the escape of a subset of B16 cells from T cell-mediated killing (**Figure 9**). To test this possibility, we examined the potential role of IFN-γ in MDA-7-mediated tumor suppression by neutralizing IFN-γ in Ad.5-*mda-7*-treated tumor bearing animals. As shown in (**Figure 8B** and **Supplementary Figure 4B** **)**, administration of anti-IFN-γ significantly protected tumor cells from Ad.5-*mda-7-*mediated effects. This observation supports a role of IFN-γ in Ad.5-*mda-7*-mediated tumor regression and supports an upregulation of PD-L1 expression in the tumor cell by IFN-γ. The upregulation of PD-L1 in surviving B16 tumor cells provides an opportunity to target this population using checkpoint inhibitors (anti-PD-L1) in combination with Ad.5-*mda-7* to determine if this will enhance antitumor efficacy. As shown in (**Figure 8C** and **Supplementary Figure 4C** **)**, Ad.5-*mda-7* significantly suppressed tumor volume and this effect was enhanced by combination treatment with anti-PD-L1. Moreover, the combinatorial effect was more robust with Ad.5-*M7S* with anti-PD-L1 than Ad.5-*mda-7* with anti-PD-L1 **(** **Figure 8C** and **Supplementary Figure 4C** **).** This effect correlated with a significant increase in IFN-γ-producing CD8^+^ T cell population **(** **Figure 8D** **)**. Ad.5-*mda-7* infection of B16 cells did not promote IFN-γ production, while Ad.5-*mda-7* infection of total spleen populations resulted in up-regulation of IFN-γ (**Supplementary Figure 5A** **)**. We further purified the T cells from spleen and treated them with either Ad.5-*null or* Ad.5-*mda-7.* Treatment with Ad.5-*mda-7* up regulated IFN-γ production in purified T cells suggesting that the regulation of IFN-γ is mediated by the tumor microenvironment and not B16 tumor cells (**Supplementary Figure 5B** **)**. These experiments support the conclusion that the antitumor effect of MDA-7/IL-24 (M7S; IL-24S) in B16 cells is partially achieved through T cell activation and the therapeutic outcome can be enhanced by the addition of checkpoint inhibitors. (**Figures 8** and **9**).

## Discussion

We currently explored two engineering approaches to enhance the therapeutic potential of the IL-10 gene family cytokine MDA-7 (IL-24) (1, 2). This involved replacement of the endogenous signal peptide (SP) of MDA-7 with an unnatural SP (insulin) to enhance secretion and introduction of a site-specific mutation in the MDA-7 protein (lysine to arginine at amino acid 122) to decrease ubiquitination and increase stability. These changes resulted in a designer cytokine, an “MDA-7 Superkine” (M7S; IL-24S), that retains cancer-selective activity while displaying increased selective antitumor activity *in vitro* toward melanoma, prostate and breast cancer cells and enhanced *in vivo* preclinical antitumor activity against human and murine melanoma and human prostate cancer.

Several strategies have been used to engineer cytokines to improve their bioactivity and antitumor efficacy. These include: enhancing affinity by modifying cytokine/receptor interfaces; altering cytokine/receptor interactions; redirecting specificity; and improving stability in serum (40). These modified cytokines are referred to as “Superkines”, e.g., alterations in IL-2 to enhance binding affinity for IL-2Rβ resulted in an “IL-2 Superkine” that improves expansion of cytotoxic T cells resulting in superior antitumor responses (23). Methods to augment half-life and improve overall bioactivity of cytokines have been achieved by chemical modifications that include PEGylation or polyethylene glycol conjugation of IL-2 (41) or IFN-α [approved by the FDA for treating melanoma (42)]. Employing a different strategy, a *de novo* computational approach resulted in the engineering of a novel cytokine, neo-2/15, that uniquely signals through shared receptors of IL-2 and IL-15 rather than their canonical receptors, IL-2Rα and IL-15Rα, resulting in preferential signaling to antitumor lymphocytes (43). Moreover, this engineered cytokine had improved therapeutic activity in murine models of melanoma and colon cancer without promoting immunogenicity (43).

Our engineering efforts focused on creating an “MDA-7 Superkine” (M7S; IL-24S) through SP shuffling and a targeted mutational strategy. SP shuffling is a routine strategy used to enhance recombinant protein production in bacterial systems (44, 45). SPs in recombinant proteins vary depending on cumulative distribution of charge/length ratio of the n-region and include 1 – 5 positively charged residues (46). The activity of the SP is also dependent on secondary structure of the encoded protein (46). Additionally, the structure of the target protein itself also plays a key role in secretion and it is possible to exchange SPs between different proteins (45, 46). Of the SPs we tested, including native endogenous MDA-7, IL-2, insulin and BM-40 SPs, the insulin SP was optimum in enhancing secretion of MDA-7 (**Figure 1**). Our data indicates that MDA-7 protein also contributes to the enhanced secretion when using the insulin SP, since replacing the endogenous IL-2 SP with the insulin SP actually decreased rather than increased secretion of IL-2 (**Supplementary Figure 1**). Barash et al. used the Hidden Markov Model (HMM) to predict the effectiveness of various human signaling peptides to direct alkaline phosphatase secretion (47). Three of our SP’s were tested using this model and the HMM bit score was quite comparable with our results with the SP from insulin > BM-40 > IL-2 in promoting MDA-7 secretion. Future studies are necessary to determine how the secondary structure of MDA-7 improves the secretory properties of our “MDA-7 Superkine” (M7S; IL-24S).

An essential component of MDA-7’s broad-spectrum anticancer activity *in vivo* is its ability to be secreted and promote an antitumoral effect on distant metastatic cancer cells, a process called “bystander antitumor” activity (10, 15, 20, 32, 48). This property of MDA-7 has been observed in many preclinical animal models as well as in a Phase I clinical trial (17, 32, 37, 48). The mechanism underlying MDA-7’s bystander effects involves an interaction of secreted MDA-7 protein with cell surface receptors resulting in synthesis and secretion of MDA-7 by these cells, through an autocrine/paracrine loop (20, 22). In cancer cells, MDA-7 induces apoptosis or toxic autophagy, whereas it elicits no apparent detrimental effect toward normal cells or tissues (6, 7). The therapeutic effects of MDA-7, including its bystander antitumor effects are dose-dependent and elevated concentrations enhance therapeutic efficacy. However, there is a fine balance that must be achieved, since a well-known harmful phenomenon called a “cytokine storm” can occur when patients encounter high levels of specific therapeutic cytokines (49, 50). In the present study, we confirm that M7S like MDA-7 also induces an antitumor “bystander” growth inhibitory effect *in vivo* in both human melanoma and prostate cancer xenografts in nude mice (**Figures 5** **-** **7**). The effect of M7S is greater than observed with wild type MDA-7 most likely because of the increased secretion of MDA-7, as well as the enhanced stability of M7S protein (**Figures 1**, **5** **–** **7**). As implied by similar promotion of PARP cleavage and promotion of cancer-selective apoptosis at lower doses of M7S vs. MDA-7, we do not anticipate that M7S will promote different biochemical and biological changes than those induced by MDA-7 in cancer cells, unless there are dose-dependent cellular/biochemical modifications (**Figures 2** **–** **4**). In a phase I clinical trial, a non-replicating adenovirus delivering MDA-7 by direct injection did not evoke toxicity (18, 19). Similarly, in the context of B16 mouse melanoma cells in syngeneic animals, no toxicity was evident in mice when Ad.5-*mda-7* or Ad.5-*M7S* was administered intratumorally (**Figure 8**). In addition, organ-specific mammary gland expression of MDA-7 in transgenic mice did not promote any toxicity, but did inhibit spontaneous and experimental tumor progression (14). Although purified MDA-7 protein is not toxic in mice when injected intravenously, in fact it induces potent antitumor activity (51), administering high doses of MDA-7 protein might provoke negative effects in humans. To avoid this potential complication it may be necessary to restrict expression and delivery of MDA-7 to tumor cells or the tumor microenvironment. This can be achieved by using conditionally replicating viruses (regulated by a cancer-selective promoter) to selectively deliver MDA-7, a Cancer Terminator Virus (*CTV*) (15, 32, 37, 48). Alternatively, one could deliver the therapeutic viruses or proteins in complement treated microbubbles administered systemically and released by focused ultrasound, the ultrasound targeted microbubble-destruction (UTMD) approach (8, 17).

Tumor cell resistance to immune-surveillance can occur through failure of tumors to present antigens to activate T cells, poor tumor immunogenicity, impaired dendritic cell maturation, downregulation of T cell activation and recruitment, and/or failure of T cell killing activity within the tumor niche. Although further studies are required, experimental data indicate that MDA-7 gene/protein can function as an effective antitumor immune stimulator, including an ability to induce a vaccine effect against different tumors, e.g., prostate and colorectal carcinomas (12–14, 52). In total, these studies support an involvement of antigen-specific T cell activation and induction of IFN-γ as initiators of MDA-7-mediated Th1 responses. Similar to preclinical observations, in clinical trials viral-mediated *mda-7* expression upregulates Th1 responses that are characterized with higher numbers of cytotoxic T cells (18, 19). To understand the impact on T cells, Zhang et al. exogenously treated both CD4^+^ and CD8^+^ T cells, isolated from cancer specimens and found that MDA-7 in a dose-dependent manner enhanced cytolytic activity of CD8^+^ T cells (as monitored by target cell death and IFN-γ expression) (52). Recently, we demonstrated that lentiviral-mediated expression of *mda-7* in tumor-sensitized T cells engenders superior tumoricidal activity in comparison with mock-modified T cells (11). Moreover, MDA-7 also directly promoted T-cell proliferation (11). Collectively, all of these mechanistic studies implicate MDA-7 in cell-mediated immunity resulting at least in part by the secretion of IFN-γ. Substantial elevation of tumor inhibitory IFN-γ might also contribute to skewing an immunosuppressive to an immunostimulatory environment. While the anti-tumor effect of IFN-γ favorably affects patient outcome, in certain contexts it can elicit immunosuppressive-effects. Insensitivity, downregulation of the MHC complex and induction of PD-L1 are likely reasons for such discrepant effects (53). In a meta-analysis of 28 studies involving more than 3000 patients it was demonstrated that expression of PD-L1 was associated with lower survival rates and poor prognosis. Of interest and consistent with these observations, MDA-7-treated tumor cells demonstrated higher expression of PD-L1. Indeed, our analysis included tumor cells that survived following multiple MDA-7 treatments, indicating that surviving tumor populations were spared from both MDA-7-mediated direct or T cell-mediated killing. Considering these findings we deduce that higher expression of PD-L1 may facilitate tumor immune evasion. Support for this conclusion comes from experiments in which anti-PD-L1 (α-PD-L1) combined with MDA-7 or M7S promotes antitumor effects against B16 cells (**Figure 8C**). As predicted, M7S significantly improved the outcome in comparison with parental MDA-7. One notable attribute of MDA-7 that contributes to its broad-spectrum anticancer properties is its ability to synergize with other therapies, including radiation (54), chemotherapy (55, 56) and antibody-based therapies (31). Our current study provides the first report that MDA-7 and to a greater extent M7S therapy is enhanced in the context of an aggressive melanoma when combined with a checkpoint inhibitor (α-PD-L1) (**Figure 8**). This combination treatment that involved multiple administrations of Ad.5-*mda-7* or Ad.5-*M7S* intratumorally with intraperitoneal delivery of α-PD-L1 resulted in decreased tumor size that was presumably T cell-mediated.

If melanoma is detected early it is readily treatable by surgery, however, once it progresses to metastasis most treatments become less effective and melanoma becomes one of the deadliest forms of skin cancer (57). Current therapeutic strategies include surgery, chemotherapy, and radiotherapy as employed in other solid tumors (58). Recent studies and clinical trials include targeted therapies, immune therapies, and combinatorial therapies depending upon the stage and location of the tumor (58, 59). α-PD-L1 checkpoint inhibitors (Pembrolizumab and Nivolumab) are now frequently used therapies for melanoma patients (29). However, although these immunotherapies are extremely effective in some melanoma patients, they are not effective in a specific subset of patients (30, 60). Various resistance mechanisms have been developed by cancer cells to evade these therapies (30). Also, initial susceptible tumors can progress into resistant tumors (61). As indicated, no currently universal therapy is available for metastatic melanoma, particularly after it has failed multiple therapies. In these contexts, developing combinatorial protocols will be necessary, which could include M7S as one of the components to treat both primary and metastatic melanoma, as well as other metastatic cancers.

To enhance the therapeutic index using M7S we plan on using adenoviruses with ‘tropism modifications’, such as serotype 5 and 3 chimeric adenoviruses (Ad.5/3), with enhanced infectivity by using multiple receptors for virus entry into target cells. An Ad.5/3 chimeric virus has proven successful in delivering *mda-7 in vivo* with enhanced activity thereby enhancing the therapy of diverse tumors (8, 37). Additional major impediments for systemically delivering adenoviruses efficiently is their destruction by the immune system and non-specific trapping in the liver. To overcome these obstacles we developed efficient systemic delivery approaches using ultrasound guided microbubble destruction (UTMD) and have successfully used this strategy to deliver adenoviruses systemically in immunocompetent animals (8, 37). In these contexts, combining Ad.5/3 delivery of M7S in combination with checkpoint inhibitors may hold significant promise for effectively treating advanced cancers including metastatic melanoma.

## Data Availability Statement

The raw data supporting the conclusions of this article will be made available by the authors, without undue reservation.

## Ethics Statement

The animal study was reviewed and approved by IACUC.

## Author Contributions

AKP, PB, SKD, and PBF conceived the study. AKP, PB, CG, SKD, X-YW and PBF designed the methodologies and experiments. AKP, SM, PB, PM, AK, and CG performed experiments and acquired data. AKP, PB, SM, SKD, LE, X-YW, JL, DS and PBF analyzed andinterpreted the data. AKP, PM, SM, and PB acquired and processed tumors and performed therapeutic studies. AKP, PB, SM, SKD, and PBF prepared the manuscript. All authors contributed to the article and approved the submitted version.

## Funding

The present study was supported in part by a grant from NIH/NCI R01 CA259599 (PBF, XYW), NIH/NCI R01 CA244993 (DS, PBF), the National Foundation for Cancer Research (NFCR) (PBF), the Human and Molecular Genetics Development Fund (LE, SKD), the VCU Institute of Molecular Medicine (PBF), a Virginia Catalyst grant (LE) and a sponsored research agreement with InterLeukin Combinatorial Therapies, Inc. (ILCT) (LE). NIH-NCI Cancer Center Support Grant P30 CA016059. PBF is the recipient of the Thelma Newmeyer Corman Chair in Cancer Research in the VCU Massey Cancer Center.

## Conflict of Interest

PBF is a co-founder and has equity in InterLeukin Combinatorial Therapies, Inc. (ILCT). VCU also has equity in ILCT. LE is the PI of a sponsored research agreement with ILCT, which is being managed by VCU.

The remaining authors declare that the research was conducted in the absence of any commercial or financial relationships that could be construed as a potential conflict of interest.

The authors declare that this study received funding from InterLeukin Combinatorial Therapies (ILCT), Inc. ILCT supported IL-24-related research through a sponsored research agreement between ILCT and VCU School of Medicine.

## Publisher’s Note

All claims expressed in this article are solely those of the authors and do not necessarily represent those of their affiliated organizations, or those of the publisher, the editors and the reviewers. Any product that may be evaluated in this article, or claim that may be made by its manufacturer, is not guaranteed or endorsed by the publisher.

## Supplementary Material

Supplementary Figure 1 Effect of Insulin signal peptide (SP) on IL-2 secretion: Canonical IL-2 SP was swapped with the Insulin SP through standard cloning. Expression constructs were transfected into Hela cells. Western blotting analysis **(A)** and ELISA **(B)** were performed to determine intracellular and secretory IL-2 protein, respectively.

Supplementary Figure 2 M7S (IL-24S) displays potent tumor growth suppression in *in vivo*. Animals were subcutaneously injected with either melanoma **(A)** or prostate cancer cell line **(B)** as described in . Tumor growth was followed over 21 days and average volume from 5 mice was plotted in each time point. *: Statistical significance (p<0.05) between control and corresponding experimental group on the 21^st^ day was presented.

Supplementary Figure 3 Effect of MDA-7/IL-24 on cancer cells: B16 and DU-145 cells were infected with the indicated Ads. 2000 VP/cell, and after 48 hr. MTT and Annexin-V assays were performed to determine proliferation **(A)** and apoptosis **(B)**, respectively. **(C)** Expression of MDA-7/IL-24 protein in B16 cells and DU-145 cells determined by Western blotting.

Supplementary Figure 4 MDA-7/IL-24 inhibits tumor growth in syngeneic animal: **(A, B, C)** Murine melanoma cells, B16 were implanted as described in (right panel), and and respective method and materials. Tumor growth was followed over 14 days and average volume from 5 mice was plotted in each time point. Statistical significance (p<0.05) between Ad.5-*null* vs Ad.5-*mda-7* **(Panel A)**, Ad.5.*mda-7* vs Ad.5-*mda-7* + Anti-IFN γ group **(Panel B)** and Ad.5-*mda-7* + Anti-PD-L1 vs Ad.5-*M7S* + Anti-PD-L1 (Panel C) was denoted by asterisk (*).

Supplementary Figure 5 Effect of MDA-7/IL-24 on IFN-γ is mediated by the tumor microenvironment, not the tumor cells: B16 cells and Splenic cell populations were infected with Ad.5-*null* or Ad.5-*mda-7* (2,000 VP/cell). Real time PCR (RQ-PCR) was done after 72 hr. **(A)**, no amplification of IFN-γ in B16 cells was observed. **(B)** T cells from spleen were infected with Ad.5-*null* or Ad.5-*mda-7* (2,000 VP/cell). Real time PCR (RQ-PCR) was done after 72 hr.
