# Supplementary figures and images for: Enhanced Cancer Therapy Using an Engineered Designer Cytokine Alone and in Combination With an Immune Checkpoint Inhibitor
Source: Front Oncol. 2022 Mar 24;12:812560. doi: 10.3389/fonc.2022.812560 (PMC8988683; doi:10.3389/fonc.2022.812560)

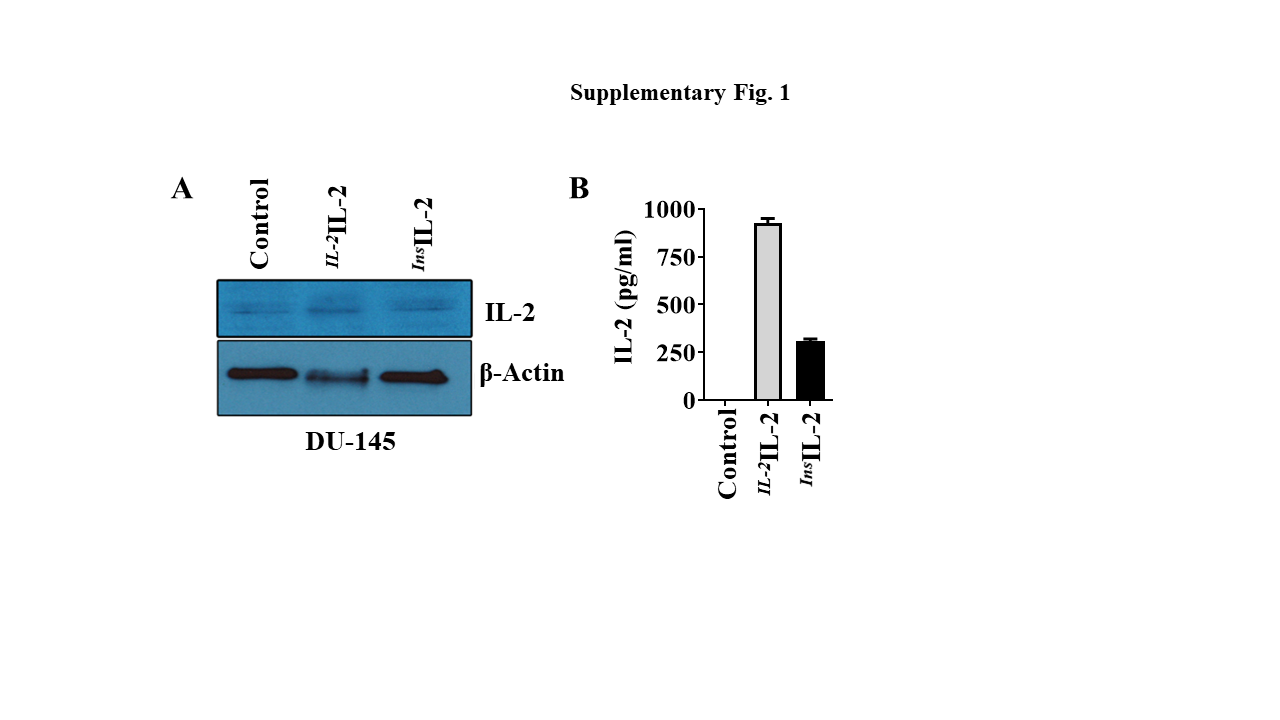

Supplement: Supplementary Figure 1 — Effect of Insulin signal peptide (SP) on IL-2 secretion: Canonical IL-2 SP was swapped with the Insulin SP through standard cloning. Expression constructs were transfected into Hela cells. Western blotting analysis (A) and ELISA (B) were performed to determine intracellular and secretory IL-2 protein, respectively. [file Image_1.tif]

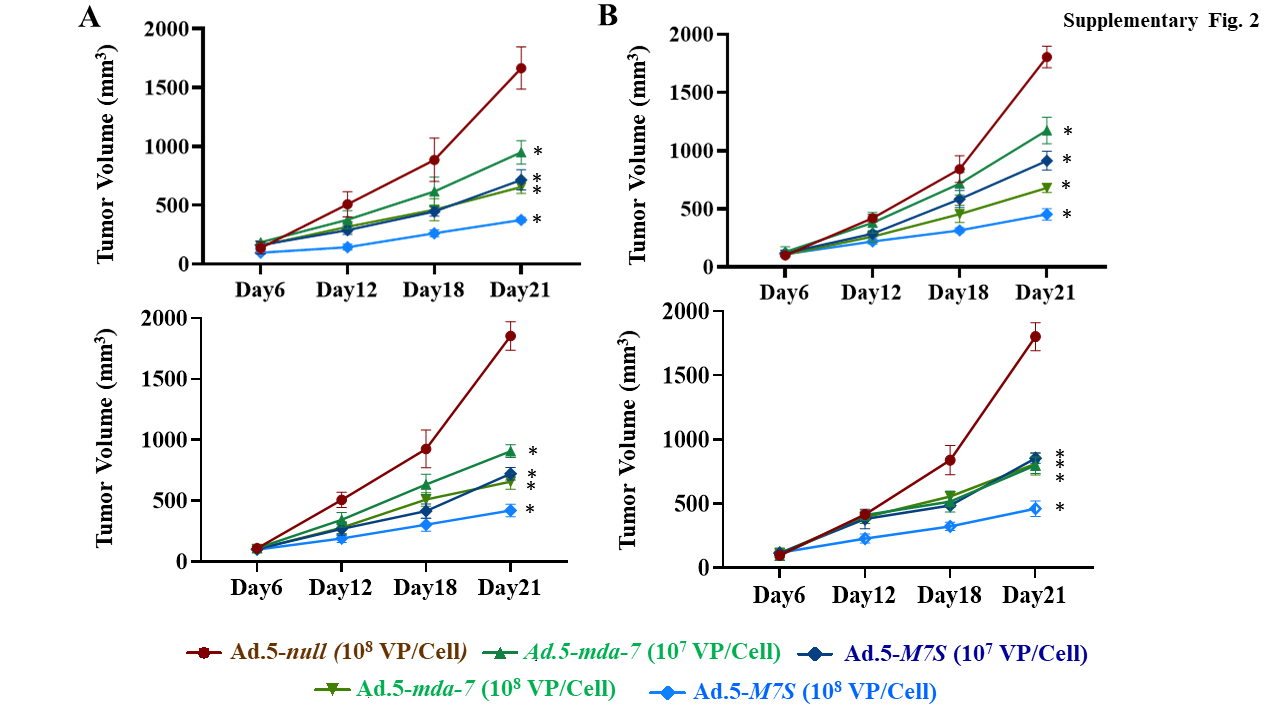

Supplement: Supplementary Figure 2 — M7S (IL-24S) displays potent tumor growth suppression in in vivo. Animals were subcutaneously injected with either melanoma (A) or prostate cancer cell line (B) as described in . Tumor growth was followed over 21 days and average volume from 5 mice was plotted in each time point. *: Statistical significance (p<0.05) between control and corresponding experimental group on the 21st day was presented. [file Image_2.tif]

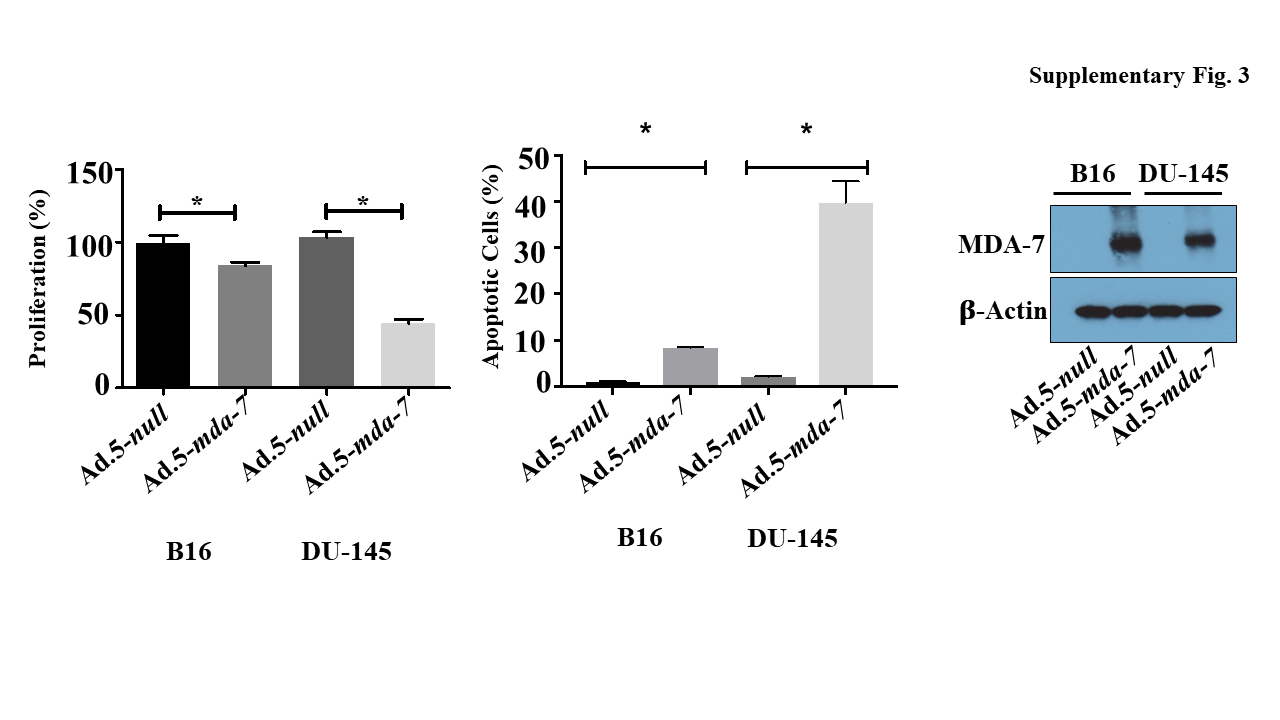

Supplement: Supplementary Figure 3 — Effect of MDA-7/IL-24 on cancer cells: B16 and DU-145 cells were infected with the indicated Ads. 2000 VP/cell, and after 48 hr. MTT and Annexin-V assays were performed to determine proliferation (A) and apoptosis (B), respectively. (C) Expression of MDA-7/IL-24 protein in B16 cells and DU-145 cells determined by Western blotting. [file Image_3.tif]

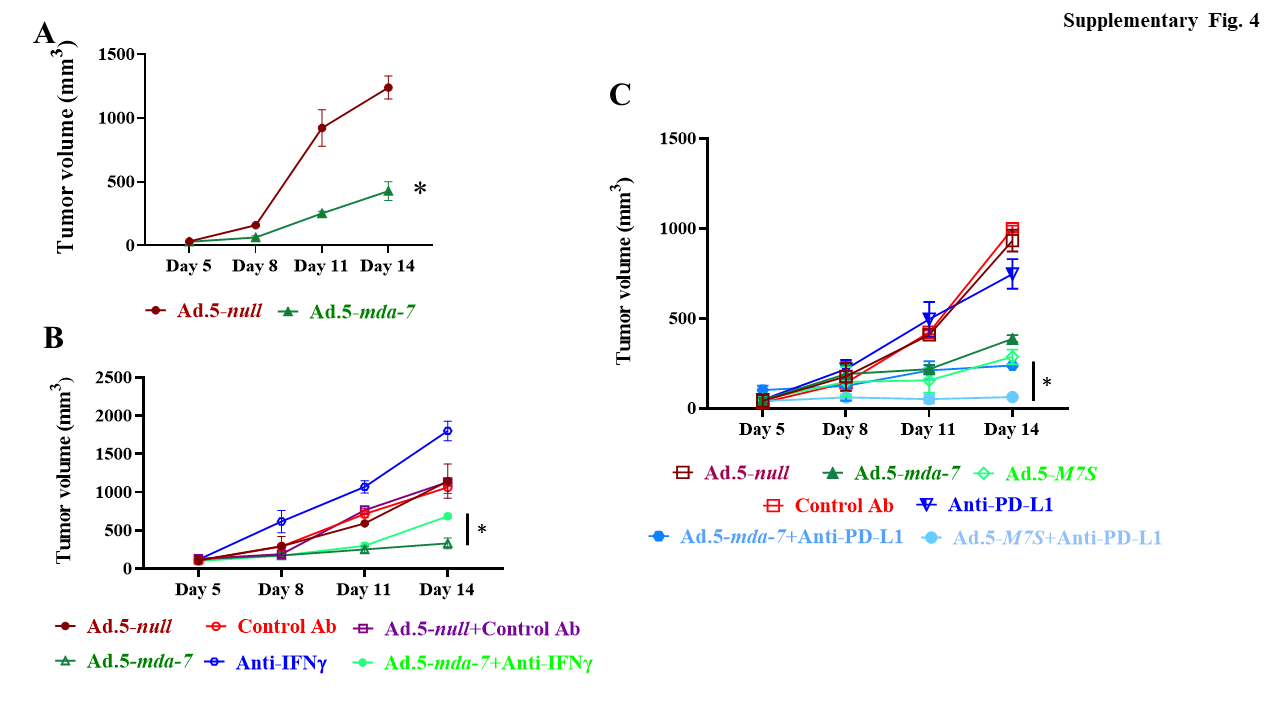

Supplement: Supplementary Figure 4 — MDA-7/IL-24 inhibits tumor growth in syngeneic animal: (A, B, C) Murine melanoma cells, B16 were implanted as described in (right panel), and and respective method and materials. Tumor growth was followed over 14 days and average volume from 5 mice was plotted in each time point. Statistical significance (p<0.05) between Ad.5-null vs Ad.5-mda-7 (Panel A), Ad.5.mda-7 vs Ad.5-mda-7 + Anti-IFN γ group (Panel B) and Ad.5-mda-7 + Anti-PD-L1 vs Ad.5-M7S + Anti-PD-L1 (Panel C) was denoted by asterisk (*). [file Image_4.tif]

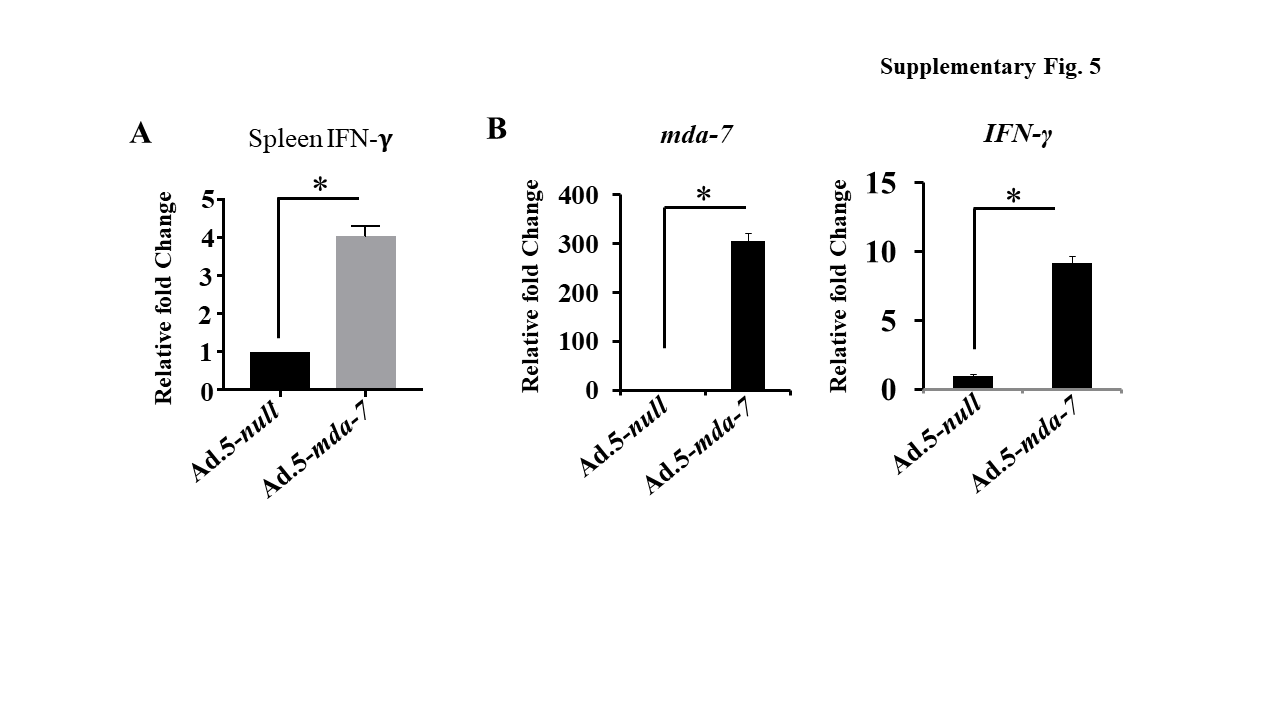

Supplement: Supplementary Figure 5 — Effect of MDA-7/IL-24 on IFN-γ is mediated by the tumor microenvironment, not the tumor cells: B16 cells and Splenic cell populations were infected with Ad.5-null or Ad.5-mda-7 (2,000 VP/cell). Real time PCR (RQ-PCR) was done after 72 hr. (A), no amplification of IFN-γ in B16 cells was observed. (B) T cells from spleen were infected with Ad.5-null or Ad.5-mda-7 (2,000 VP/cell). Real time PCR (RQ-PCR) was done after 72 hr. [file Image_5.tif]
